# Structural changes of a protein extract from apple with polyphenoloxidase activity obtained by cationic reversed micellar extraction induced by high-pressure carbon dioxide and thermosonication

**DOI:** 10.1038/s41598-019-50209-w

**Published:** 2019-09-24

**Authors:** A. E. Illera, S. Beltrán, M. T. Sanz

**Affiliations:** Department of Biotechnology and Food Science (Chemical Engineering Section), University of Burgos, 09001 Burgos, Spain

**Keywords:** Chemical engineering, Chemical engineering

## Abstract

Polyphenoloxidase from apple was extracted and further concentrated by cationic reversed micellar extraction. Previous to reversed micellar extraction a crude protein extract was obtained using AG2-X8 as adsorbent of phenolic compounds and the detergent Triton X-100. Forward and backward extraction conditions were optimized by using dodecyl trimethyl ammonium bromide as surfactant in the organic phase. Optimization was carried out to obtain the highest value of PPO activity recovery and the purification fold at the different experimental conditions. Under the optimum extraction conditions, PPO activity recovery was 99% and purification fold reached a value of 17, showing that reversed micellar extraction was a good technique as a first step to concentrate on a targeted enzyme. After removing some impurities by centrifuge ultrafiltration, the protein extract with PPO activity was treated by pressurized carbon dioxide and thermosonication achieving residual PPO activity values of 16 ± 3 and 9 ± 1%, respectively. Quenching experiments by iodide performed in the non-treated extract and in the treated extracts revealed conformational changes of this protein fraction reflected in the greater exposure of the fluorophore to the quencher.

## Introduction

Consumers demand fresh and minimally processed products without chemical preservatives. Traditionally, thermal treatments have been employed to avoid microbial spoilage and achieve the inactivation of some enzymes that are responsible for unfavorable changes on foods. But, thermal treatments can cause undesirable changes in physical and nutritional parameters. Therefore, the food industry is continuously searching for new processing and preservation methods. Some of these technologies include the use of high pressure, such as high hydrostatic pressure (HPP) or high pressure carbon dioxide (HPCD), electric fields, and ultrasound (US) among others^1^. In previous works, the effect of HPCD and thermosonication (TS) treatments on the activity of polyphenoloxidase (PPO) and pectinmethylesterase (PME) from *Golden Delicious* was studied. PPO is one of the enzymes involves in the enzymatic browning on apple products. At 20 MPa, 45 °C for 60 min, HPCD treatment could reduce PPO activity reaching values of 13.4% of residual activity^2^. However, application of moderate temperatures (50–67 °C) was necessary when using the US technology to reach low residual activity of PPO, 71% and 5% at 50 and 67 °C, respectively during 20 min of continuous TS^3^.

The knowledge of the mechanism of enzyme inactivation is of great importance for the optimization of these new preservation methods. Mechanism for enzyme inactivation by HPCD is not still clear. Different mechanisms have been reviewed by Hu *et al*.^4^ including conformational changes of the enzyme caused by the pressurized CO_2_ or molecular mechanisms associated to the formation of complexes of the CO_2_ with the protein. Recently, Illera *et al*.^5^ determined CO_2_ solubility in different fruit juices to understand the role of CO_2_ on enzyme inactivation mechanism. Regarding enzyme inactivation by TS, different mechanism can play an important role on enzyme inactivation of enzymes, mainly shear forces and the formation of localized hot spots as well as the sonolysis of water that originates the formation of free radicals^6^. Characterization of PPO structure in its original matrix is complicated since many other compounds present in the juice can interfere (sugar, pectin, polyphenols and other proteins). In this work, PPO from ‘*Golden Delicious*’ apple has been concentrated by reversed micellar extraction (RME) to obtain a protein extract with PPO activity that has been later treated by two different non-thermal technologies, HPCD and TS.

RME has been selected as separation and concentration method of PPO from *Golden Delicious* apple, based on the good results obtained by Imm and Kim^7^ on partial purification of PPO from apple skin by RME by using a cationic surfactant^7^. In a RME extraction process, a target protein in an aqueous phase is transported to the organic phase composed by reverse micelles (forward extraction). Consequently, the protein must be liberated into a fresh aqueous phase stripping solution (backward extraction)^8^. The distribution coefficient of the protein between the two phases is determined by different parameters of the aqueous and organic phase such as pH, ionic strength, type of salt, the surfactant and co-surfactant employed, as well as by changes in temperature^9^. RME is an attractive extraction method to recover different biochemical compounds without losing their native activity. In addition, it offers a low interfacial tension, it can operate easily in a continuous mode since it is a technique easy to scale-up^8^. However, pure enzymes cannot be isolated by RME and this technique must be considered as a first separation step.

In this work, RME has been optimized as a first step for PPO purification from apple, as proposed by Imm and Kim^7^ for PPO from apple skin. RME process was optimized to obtain the highest value of PPO activity recovery and purification fold. The optimum extract was treated by two different non-thermal technologies, HPCD and TS, to further determine conformational changes on the protein extract. Although RME process was optimized in terms of, activity and purification fold of PPO, peroxidase activity, POD, as one important enzyme in discolouration on fresh-products, was also determined before and after both non-thermal treatments. Fluorescence spectroscopy, before and after treatments, was used to determine global changes in the tertiary structure of the protein extract by quenching experiments.

## Materials and Methods

### Crude PPO extract preparation

The extraction procedure of PPO from *Golden delicious* apple (pH = 3.76 ± 0.01, °Brix = 10.83 ± 0.06), was performed according to Zhou *et al*.^10^ for apple skin with some modifications. The anion exchange resin AG2-X8 (Bio Rad Laboratories) was used as adsorbent of phenolic compounds during the extraction to avoid PPO inactivation during extraction. Additionally, the non-ionic detergent Triton X-100 was employed to facilitate solubilization of the membrane bound-PPO^11^. The unseeded apple was grounded with liquid nitrogen in a glass blender to decompose cell membranes. Sodium phosphate buffer, pH = 7.2, with different amounts of AG2-X8 (0 to 2 g resin/g apple) and Triton X-100 (from 0 to 0.15 wt.%) was mixed with the grounded apple at the ratio 1.7 mL of buffer/g apple, as the optimum ratio determined by Rocha and Morais^11^ for PPO extraction from apple (*cv. Jonagored*). The mixture was kept 1 h in the fridge at 4 °C. Afterwards, the protein extract was filtered and centrifuged for 30 min at 5000 g and 4 °C. In the supernatant, PPO activity was determined. The crude extract with the highest PPO activity was further concentrated by RME.

### Reverse micellar extraction

#### Forward extraction

Different parameters affect the distribution of a protein between the organic phase and the aqueous, among them, the pH, the ionic strength or the type of salt^9^. The pH was adjusted by using two different types of buffers to obtain the aqueous extract. Low pH values, pH = 4–6, were obtained by using a citrate buffer, 100 mM, while higher pH values, pH = 7–8, were obtained by using a sodium phosphate buffer, 100 mM. The effect of the ionic strength was studied by varying the concentration of KCl from 0 to 100 mM. The organic phase was constituted by the cationic surfactant dodecyl trimethyl ammonium bromide (DTAB), at different concentrations (50–200 mM) dissolved in isooctane mixed with hexanol as co-surfactant at the ratio 5:1 (v/v), according to Imm and Kim^7^. When using a cationic surfactant, very small micelles are formed and the addition of an alcohol, acting as a co-surfactant, makes the micelles grow improving water uptake and solubility capacity of the organic phase^8,12^. The first step in the RME process was carried out by mixing thoroughly equal volumes of the organic phase and the crude extract for 20 min at 4 °C, at different pH values, from 4 to 8, and different ionic strength values by varying KCl concentration from 0 to 100 mM. Aqueous and organic phases were separated by centrifugation at 4 °C and 2800 g. Forward extraction was also carried out at three different temperatures from 4 to 25 °C, since protein solubilization is affected by changes in temperature^9^. The extracted protein in the organic phase was collected for the backward extraction.

#### Backward extraction

To recover the protein extracted in the reversed micellar phase, equal volumes of the organic phase and of an aqueous stripping solution consisted in sodium phosphate buffer (pH = 6) with 1 M KCl and 10% v/v ethanol were mixed vigorously^7^. The use of alcohol, such as ethanol, in the second step of the RME process helps to weaken the hydrophobic interactions between the micelles created by the surfactant and the solubilized protein, especially when the extraction conditions selected for the backward extraction are not enough to release the protein^8^. Separation of organic and stripping aqueous phases was carried by centrifugation at 4 °C and 2800 g for 90 min. This stripping solution was used in all the experiments where forward extraction was optimized. Forward and backward experiments were performed at the same extraction temperature, varying this parameter from 4 to 25 °C. KCL concentration in the aqueous stripping solution vas varied from 0.05 to 1 M to study the effect of the ionic strength in the stripping solution. Backward extraction was also carried out in the absence of ethanol in the stripping solution to consider if only a high salt concentration was enough to disrupt the interactions of the protein with the micelles of the organic phase. The RME process was characterized by determining the recovery of PPO activity and the purification fold in the stripping aqueous solution.

#### Centrifuge ultrafiltration

The optimum extract obtained by RME with the highest activity recovery and purification fold was treated by centrifuge ultrafiltration by using Amicon® Ultra centrifugal filters (15 mL, NMWL:3000) with a regenerate cellulose membrane of the filters that allowed a high PPO activity recovery. The final retentate volume was brought to the initial treated volume with the same sodium phosphate buffer as the one used in the backward extraction.

### Parameters to determine the efficiency of the reverse micellar process

#### Protein content in the aqueous phase

Total protein content was determined in the aqueous crude extract and in the aqueous stripping solution after backward extraction, by using the kit *RC DC*^TM^ (Bio Rad Laboratories). This assay is based on the Lowry protocol and it avoids interferences in the protein determination due to the presence of reducing agents and detergents such as Triton X-100. A calibration curve was done by using bovine serum albumin as standard.

#### Determination of PPO activity

The protocol to determine the activity of PPO was explained in detail by Illera *et al*.^2^ Briefly, a 0.05 M catechol (Sigma Aldrich) in a 0.1 M phosphate buffer (pH 6.5) solution was used as substrate for PPO. 100 µL of protein extract were mixed with 2.9 mL of the catechol solution and catechol oxidation was followed at 30 °C by spectrophotometry at 420 nm (Jasco V-750 spectrophotometer). The initial linear part of the reaction curve was taken to determine PPO activity taking into account that one unit of PPO activity was defined as the amount of enzyme required for 0.001/min absorbance increase under the reaction conditions^7^.

#### Determination of peroxidase activity

Peroxidase (POD) activity was determined spectrophotometrically according to Soysal *et al*.^13^. Reaction started when mixing 2.7 mL of 0.01 M acetate buffer (pH 5), 0.1 mL of 0.1% (v/v) H_2_O_2_, 0.4 mL of 0.05% (w/v) o-dianisidine in methanol and 0.1 mL of protein extract. Absorbance was measured at 460 nm during 120 seconds by using a Jasco V-750 spectrophotometer. The POD activity was only observed qualitatively by showing the initial linear slope of the reaction curve and no numerical value for POD activity was reported in this work.

#### PPO activity recovery and purification fold

The percentage of the recovery of PPO activity (activity recovery, AR) is defined as

1 $$AR( \% )={A}_{b}/{A}_{i}\cdot 100$$

Purification fold (PF) is defined as

2 $$PF=({A}_{b}/{C}_{b}{V}_{b})\cdot ({C}_{i}{V}_{i}/{A}_{i})$$

where A_i_ and A_b_ are the PPO activity in the initial crude protein extract and backward extraction aqueous phase, respectively, V_i_ and V_b_ (mL) are the volume of the initial crude protein extract and backward extraction aqueous phase, respectively and C_i_ and C_b_ are the concentration of protein (mg/L) in the initial crude protein extract and backward extraction aqueous phase, respectively^7,14^.

### Treatment of PPO extract by HPCD and thermosonication

The protein extract with the highest recovery of PPO activity and purification fold, was treated by HPCD and TS. Residual activity after treatment was determined and possible conformational changes were verified by fluorescence spectroscopy.

#### HPCD treatment

The experimental set up has been previously described in detail^2^. In a HPCD experiment, 40 mL of protein extract were charged in a stainless steel high pressure batch reactor (80 mL) that it is submerged in a thermostatic water bath to control the temperature. Pressurized CO_2_ was introduced into the enzyme solution through a 10 µm filter by using a high pressure syringe pump (260D Teledyne ISCO). HPCD treatment was carried out for 60 min at 20 MPa and 45 °C, based on previous inactivation studies of PPO from *Golden Delicious* cloudy apple juice^2^.

#### Thermosonication

The ultrasound equipment used in this work was a 750 W ultrasonic processor (Sonics and Materials^TM^) with a 13 mm probe operating in a continuous mode. 80 mL of protein extract were treated for 20 min at 100% amplitude which was equivalent to a power density of 1.36 W/mL. Medium temperature treatment was 64 °C^3^.

Residual PPO activity achieved after HPCD and thermosonication treatments, was determined as the ratio between PPO activity after and before treatment.

### Fluorescence spectroscopy

The tertiary structure of the protein extract before and after HPCD and TS treatment was determined by fluorescence spectroscopy (FLS980 photoluminescence spectrometer). The non-treated and treated extract was excited at a λ_em_ = 280 nm, recording the emission spectra from 290 to 400 nm^15^. The emission and excitation slits were set at 2 and 1 nm, respectively.

Conformational changes of protein extract with PPO activity induced by HPCD and TS treatments were confirmed by tryptophan fluorescence. The quencher consisted in a potassium iodide solution (KI, 2 M) with 0.1 M sodium thiosulfate^16^, to prevent the formation for triiodide. Different aliquots of the potassium iodide solution were added to the protein extract. After each addition, the fluorescence spectra were registered. The quenching process of the tryptophan fluorescence was evaluated by using the Stern-Volmer equation:

3 $${I}_{o}/I=1+{K}_{SV}Q$$

where I_o_ correspond to the fluorescence intensity in the absence of the quencher, I are the fluorescence intensities at the different concentrations of the quencher, Q, and K_SV_ is the Stern-Volmer constant. This constant describes the accessibility of the quencher to the fluorophore (tryptophan), being more affected when the tryptophan residue is located on the protein surface^17^.

### Statistical analysis

The software Statgraphics Centurion (Version 17.3.02, 64-bit) by Statpoint Technologies, Inc. was used to carry out the statistical analyses. The Tukey’s honestly significant difference (HSD) method at p-value ≤ 0.05 was selected to determine the significance of the differences. An ANOVA test was applied to test if there was any statistical significance of the slope in the equation of Stern Vollmer, K_SV_ for the untreated protein extract and HPCD and TS treated extract.

## Results and Discussion

### Optimization of crude PPO extract

AG2-X8 and Triton X-100 concentration was varied in the range from 0 to 2 g of resin/g of apple, and 0 to 0.15 wt.%, respectively. Table 1 presents the PPO activity for the crude extracts for all the experiments performed. When neither resin nor Triton X100 was employed in the extraction, the PPO activity of the crude extract presented the lowest value. PPO extraction significantly improved with the presence of the resin due to its ability to adsorb endogenous phenolic compounds from the extraction medium^10^. A ratio of 0.5 g resin/g of apple was found to be the optimum, since higher ratios did not bring a further increase in the activity of PPO.

**Table 1:** Influence of AG2-X8 and Triton X-100 concentration on PPO activity of the crude extract by using phosphate buffer, pH = 7.2.

g resin/g apple	% Triton X-100	U/g apple
0	0	326 ± 30^a^
0	0.15	1214 ± 134^b^
0.5	0.15	1900 ± 90^c,d^
1	0.15	1821 ± 20^c^
2	0.15	1379 ± 101^b^
0.5	0	1205 ± 119^b^
0.5	0.02	1819 ± 103^c^
0.5	0.05	1920 ± 105^c,d^
0.5	0.1	2166 ± 74^d^
0.5	0.15	1900 ± 90^c,d^

Different letters in the same column indicate significant differences by applying the Tukey’s honestly procedure.

At a ratio of 0.5 g resin/g of apple, PPO activity sharply increased with the addition of 0.02 wt.% of Triton X-100, reaching a plateau up to the highest concentration essayed in this work, 0.15 wt.%. Janovitz-Klapp *et al*.^18^ also found that PPO activity increased sharply up to 0.5 wt.% of Triton X-100, reaching a plateau in the PPO extraction from ‘*Red Delicious*’ apple. Based on these results, further extractions were carried out by adding 0.5 g resin/g of apple and 0.02% Triton X-100 to the corresponding extraction buffer.

PPO has been recognized as the main enzyme related with the enzymatic browning of apple; however, POD enzymes can also contribute to the discoloration in fresh-cut products. Therefore, although crude extract optimization was done considering PPO activity, POD activity was also determined in the crude extract, observing some POD activity.

### Concentration by reverse micellar extraction

#### Forward extraction

**Figure 1:**
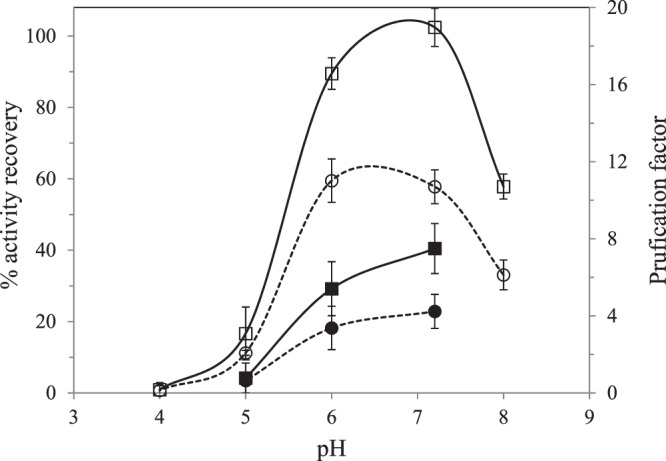
Effect of pH of the initial aqueous phase on activity recovery (□, ■) and purification fold (ο, ●) of PPO. Solid symbols correspond when no Triton X-100 was added to obtain the initial crude extract.

Effect of pH: Figure 1 shows a strong influence of the pH on the AR and PF, with no KCl was added to the extraction medium and at a DTAB concentration of 100 mM. Buffer extraction media at pH values of 6 and 7.2 yielded the highest purification factor, although slightly higher values of activity recovery were obtained at pH = 7.2.

For cationic surfactants, such as DTAB, pH values above the isoelectric point of the targeted protein favors its solubilization in the reversed micellar phase^19^. According to the literature, the reported isoelectric point of PPO from ‘*Red Delicious*’ apple was 4.5–4.8^7^. At pH above its isoelectric point, PPO presents a net negative charge that favors electrostatic interactions with the cationic surfactant. In any case, it must be highlighted that not only electrostatic interactions play an important role on protein solubilization but also hydrophobic interactions^9^. Rocha *et al*.^11^ studied PPO extraction from apple (*cv. Jonagored*) with addition of 2% PVP and 0.25% Triton X-100 to the extraction buffer. These authors reported two maxima in the extraction pH vs. activity curve, at 5.0 and 7.5 at 20 °C, corresponding to PPO from chloroplasts and from mitochondria, respectively. However, these authors found that the peak at pH values of 5.0 was lower than at 7.5 when cathecol was the substrate; concluding that, the detergent could favor mitochondrial PPO extraction. On the contrary, previous studies reported that Triton X-100 preferentially extracted apple PPO from chloroplasts than from the mitochondria. Rocha *et al*.^11^ suggested that the use of detergents could affect either the cytoplasm or the mitochondrial enzyme.

At pH above 7.2, both, PPO AR and PF dropped since enzyme inactivation could take place in some extent. Imm and Kim^7^ found an optimum value for pH of 6 for PPO extraction from apple skin by RME with DTAB, although activity recovery was less than 50% with purification fold slightly above 10. These authors, at pH = 7, obtained similar PF, but the AR was less than 20 wt.%. Although a similar protocol was followed to the one proposed, higher AR values were obtained. These differences could be attributed to the way the crude extract was obtained. Imm and Kim^7^ employed double-deionized water containing the resin as extracting medium to obtain the crude extract with no addition of Triton X-100. As described in the previous section, the presence of Triton X-100 increased the PPO activity in the crude extract. To analyze the effect of Triton X-100 in the RME process, experiments were carried out in the pH range from 5 to 7.2 without adding Triton X-100 to obtain the crude extract. Figure 1 shows that AR and PF were significantly lower when no Triton X-100 was added to the extraction medium. Zhou *et al*.^10^ also reported a positive effect on AR from apple peel when using the detergent Triton X-100, attributing this behavior to the disruption of chloroplast membranes, facilitating the release of different PPO fractions^10^.

**Figure 2:**
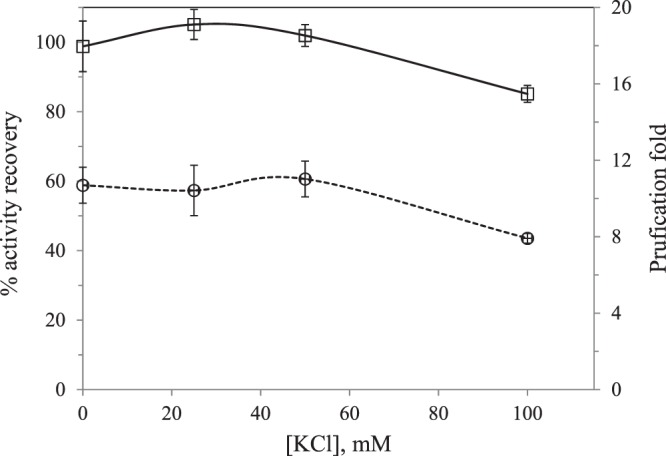
Effect of ionic strength in the forward extraction on activity recovery (□) and purification fold (ο) of PPO.

Effect of ionic strength: It has been shown that the buffers employed in this work can supply enough of the electrolytes to achieve the minimum value for reversed micelles formation with no phase separation. In the literature^9^, it has been reported that high ionic strength values lead to small micelles formation and it could reduce the interactions between the hydrophilic biomolecules and the polar group of the surfactant molecule. Therefore, the effect of higher values of the ionic strength by adding increasing amounts of KCl to the extraction medium was studied to determine the maximum salt concentration that can be present in the extraction medium to avoid a decrease in the protein solubilization in the organic phase. Therefore, forward extraction was performed at the optimum pH of 7.2 and 100 mM of DTAB under different concentrations of KCl, from 0 to 100 mM (Fig. 2).

The AR and PF did not change up to 50 mM of KCl, while a decrease was observed at the highest concentration of KCl essayed in this work, 100 mM. At this concentration, the protein solubilization in the organic phase decreased. At a certain value of the ionic strength in the aqueous phase, electrostatic interactions between protein and surfactant decreased due to the Debye screening effect caused by the presence of mobile charge carriers^9^. Certain ionic strength is needed to form reverse micelles and phase separation, in this work, when no KCl was added, a good performance was observed. This could be attributed to the use of a buffered aqueous solutions that can supply enough ionic strength^9^. Imm and Kim^7^ obtained also the best results when no KCl was added to the extraction medium, with 43% of activity recovery and a value of 13 for the purification fold.

**Figure 3:**
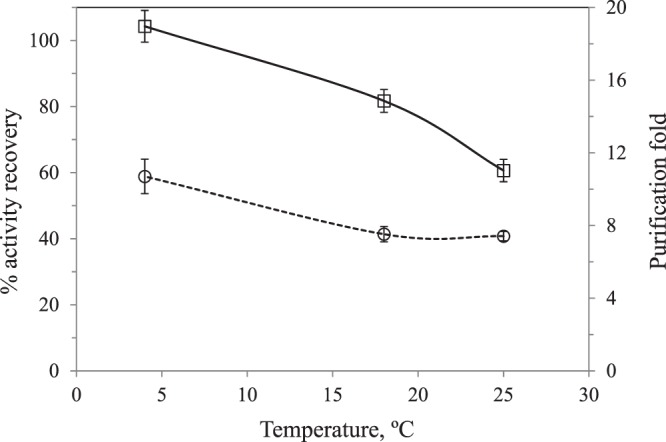
Effect of extraction temperature on activity recovery (□) and purification fold (ο) of PPO.

Effect of temperature: Reversed micellar extraction was carried out at three different temperatures, 4, 18 and 25 °C, at pH = 7.2, DTAB concentration of 100 mM and no KCl added to the aqueous phase. Figure 3 shows that when temperature increased, both, PPO AR and PF decreased.

In this regard, the effect of temperature on RME is still unclear, since phase behavior and mass transfer phenomena are involved. According to Liu *et al*.^14^, an increase in temperature improves mass transfer and therefore the time needed for protein transfer through the interface decreases, but also, an increase in temperature leads to a decrease in the partition coefficient. Dekker *et al*.^20^ in the study of the separation of α-amylase by reversed micellar phase using trioctylmethylammonium chloride (TOMAC) observed that by increasing the temperature, the amount of solubilized water decreased. These authors, proposed a method of desolubilization of the protein by an increase in the temperature for the backward extraction, avoiding the use of a second aqueous phase. On the other hand, an increase in the extraction temperature, could cause enzyme inactivation. However, during the extraction time, at the working temperatures of this work, it was proved that no PPO inactivation took place. According to Fig. 3, the decrease of the protein partition coefficient with an increase of temperature was dominant. The lowest temperature essayed in this work, 4 °C, was enough to facilitate mass transfer during the 20 min of extraction time fixed in this work. Chen *et al*.^21^ reported a maximum at 15 °C in the chitonase activity recovery by RME with AOT in isooctane. These authors reported that higher values of temperature might have loosened the structure of the reversed micelles, hindering the migration of the enzyme from the aqueous to the organic phase to enter into the micelles^20^.

**Figure 4:**
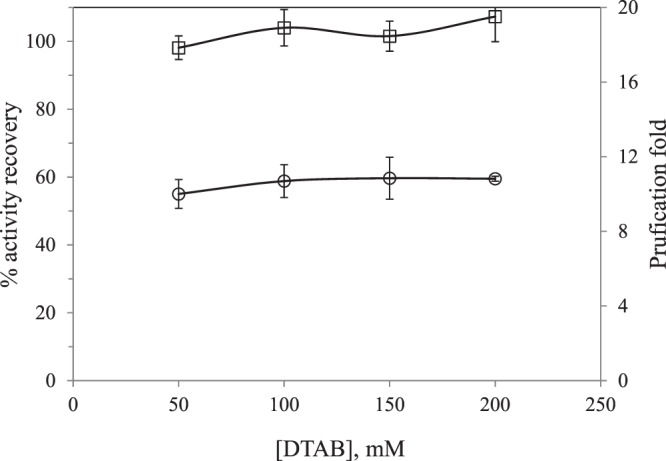
Effect of DTAB concentration in the forward extraction on activity recovery (□) and purification fold (ο) of PPO.

Effect of surfactant concentration: The nature and concentration of surfactant are important factors that determine enzyme solubilization. According to Imm and Kim^7^, DTAB was found to be an adequate surfactant to extract PPO from apple skin. In this work, DTAB was the only surfactant essayed and its concentration was varied from 50 to 200 mM at 4 °C, pH = 7.2 and no addition of KCl. In the literature, it was observed that by increasing the surfactant concentration protein solubilization in the organic phase is favored^9^. But for the DTAB concentration values of this work, no significant change on PPO AR and PF was observed (Fig. 4). However, Imm and Kim^7^ observed a maximum AR around 40% and PF of 12 at DTAB concentration of 100 mM. In this work, 50 mM seems to be enough to assure total protein mass transfer. This could be attributed to the presence of Triton X-100 that could contribute to solubilize proteins. A negative effect at the highest concentration, 200 mM, was not observed and probably, at this concentration, micellar interaction was still not taking place, showing that the electrolytes of the aqueous buffer solution still provided enough ionic strength to reduce electrostatic repulsion between the surfactants molecules.

#### Backward extraction

**Figure 5:**
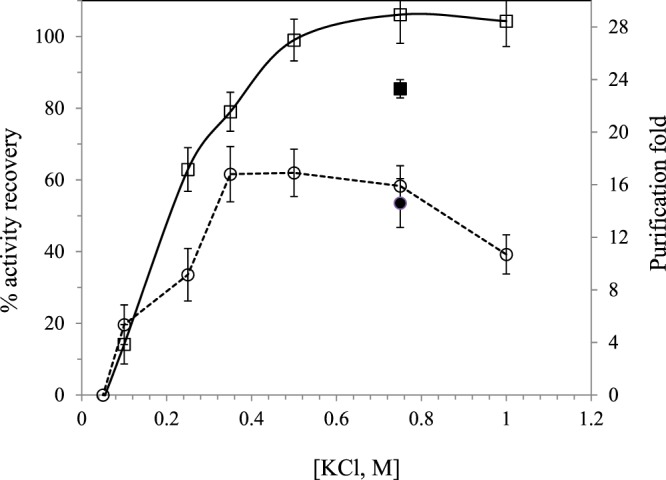
Effect of KCl concentration in the stripping solution of the backward extraction on activity recovery (□) and purification fold (ο) of PPO. Solid symbols correspond to backward extraction performed in the absence of ethanol.

To analyze the effect of some parameters of the backward extraction step, parameters of the forward extraction were fixed at 4 °C, pH = 7.2 and no addition of KCl. 100 mM DTAB was fixed since the effect of other extraction parameters in the forward extraction was studied at this concentration, although 50 mM was found to be the optimum surfactant concentration, considering the optimum value as the lowest limit studied in this work to maximize the protein transfers to the micellar phase. Backward extraction was carried out with a stripping solution of sodium phosphate buffer (pH = 6) with and ethanol concentration of 10% v/v^7^. One of the most important parameters in the backward extraction is the value of the ionic strength in the stripping solution. The effect of this variable was studied by varying the KCl concentration in the range from 0.05 to 1 M (Fig. 5). An ionic strength around 0.5–0.75 M of KCl was needed to achieve high AR and PF factor. At 0.5 M of KCl, AR and PF were 99 ± 6% and 17 ± 2, respectively. Lower KCl concentrations were not enough to break the interactions between the solubilized protein and the micelles, and both AR and PF sharply decreased when decreasing KCl concentration. At 1 M KCl, although activity recovery is still high, purification fold decreased down to 11 ± 2. Soni and Madamwar^22^ found that at 0.2 M KCl, acid cellular phosphatase activity recovery from fermentation broth was maximum, observing also a decreasing trend in back transfer protein recovery by further increasing ionic strength. At 0.75 M KCl, backward extraction was also carried out in the absence of ethanol (Fig. 5). Although purification fold factor was similar in the absence or presence of ethanol, activity recovery was significantly lower when no ethanol was present in the stripping solution. According to the literature^8^, the presence of alcohol helps to weaken the strong hydrophobic interaction of the protein with the surfactant favoring protein release^8^. Other studies, presented similar results. Yu *et al*.^23^ studied the backward extraction of a yeast lipase after the previous forward AOT RME in isooctane at ethanol concentrations in the range from 0 to 6% by volume with 0.5 M of KCl at pH 8. When ethanol was not added, the lipase could not be recovered. The lipase activity recovery was around 40% in the presence of 1% of ethanol, while it increased up to 68% for an ethanol concentration of 3%, with no improvement at higher ethanol concentrations.

In the literature, PF of the same order as the one obtained in this work, has been reported for RME. Imm *et al*.^7^ reported a purification fold of 13 (with 71% of activity recovery) for PPO recovery from apple skin by using DTAB. A purification factor of nearly 10 has been reported by Liu *et al*.^14^ in the study of concentration of nattokinase from fermentation broths by AOT/isooctane system. A maximum purification factor value of around 32 has been even reported from lysozyme extraction from freeze-dried egg-white of hen eggs by using cetyldimethylammonium bromide (CDAB)^12^.

In any case, PF values obtained in this work by RME, were lower than the values reported by Marrufo-Hernández *et al*.^24^ for PPO purification from ‘*Golden Delicious*’ apple with values of 30 and higher than 300 by fast protein liquid chromatography with a column packed with phenylsepharose and subsequent ion-exchange purification step, respectively. These results showed that RME is an easy technique to perform a first concentration step, but further purification steps are needed to isolate the enzyme.

#### Centrifuge ultrafiltration

Figure 1 showed that higher PPO AR and PF were achieved by using Triton X-100 in the extraction medium. However, further purification with ammonium sulphate fractionation was not possible when Triton X-100 was in the medium observing no precipitate. This fact was also reported by Zou *et al*.^10^ in the extract of PPO from apple peel when using Triton X-100, attributing this behavior to the interference of green pigments co-extracted and excess of the detergent.

Centrifuge ultrafiltration by using Amicon^®^ Ultra centrifugal filters (15 mL, NMWL:3000) was proposed for removing small molecules, such as salt molecules, and the detergent Triton X-100. A further PPO purification fold factor of 2 ± 0.4 was achieved and PPO activity recovery was higher than 95%. However, further purification with ammonium sulphate fractionation was still unsuccessful. According to the membrane distributor company (Millipore Corporation), at concentrations above 0.01%, Triton X-100 forms micelles resulting in aggregation of the detergent, affecting the amount that removed by centrifugal ultrafiltration. 100000 MWCO would be required to remove the detergent effectively; however, this MWCO membrane will not retained the PPO. In any case, centrifuge ultrafiltration will help to remove the salt form the aqueous phase, before HPCD and TS treatments.

After the centrifuge ultrafiltration process POD activity was not observed, although some POD activity was observed in the crude extract.

### Effect of HPCD and thermosonication on protein extract with PPO activity

Enough amount of protein extract with high PPO activity was obtained by RME to be later treated by HPCD and thermosonication. Forward extraction conditions were the following, 4 °C, pH = 7.2, 100 mM DTAB and no addition of KCl to the extracting medium. Backward extraction conditions were 4 °C, pH = 6, 0.5 M KCl and 10% v/v of ethanol.

Conditions for HPCD treatment were selected based on previous work on PPO inactivation by HPCD^2^, 45 °C and 20 MPa for 60 min. After treatment, PPO residual activity value was 15.5 ± 2.5%. When protein extract was treated by TS in a continuous mode during 20 min at 64 °C a PPO residual activity value of 8.7 ± 0.8% was obtained. Although POD activity was not found after centrifuge ultrafiltration, POD activity was also measured after HPCD and TS treatments, founding no activity according to the previously reported method.

In previous works^2,3^, PPO residual activity values of 13.4% and 16.4% were reached by treating cloudy apple juice from *‘Golden Delicious’* by HPCD and TS treatments, respectively, at the same working conditions. The lower PPO residual activity value observed by TS could be due to the presence of other food components that could protect the enzyme^25^.

Although inactivation mechanisms by new non-thermal technologies are still not clear, different theories have been proposed. Possible enzyme inactivation mechanisms by HPCD have been reviewed by Hu *et al*.^4^ such as pH lowering due to CO_2_ dissolved in the hydration layer that would form carbonic acid, that will yield bicarbonate, carbonate and H^+^ ions or inhibitory effects of molecular CO_2_. Regarding TS, inactivation of enzymes by sonication is attributed to different physical and chemical effects^26^. The different enzyme inactivation mechanisms would lead to different inactivation degree due to different structural changes after both treatments. In this work, changes on the tertiary structure due to relocation of tryptophan residues was studied by performing fluorescence spectroscopy analysis.

#### Conformational changes of protein extract with PPO activity after HPCD and thermosonication

The fluorescence properties of non-treated and HPCD and TS-treated protein extract were investigated through intrinsic fluorescence and quenching studies. The λ_max_ for the non-treated extract was 318 nm. According to the literature, values of λ_max_ < 330 nm show that tryptophan (Trp) is being buried and in a “nonpolar” environment^27^. After HPCD and TS treatment of the protein extract, slightly changes were observed in λ_max_, with a blue shifted of TS-treated protein extract (λ_max_ = 317 nm) and a red shifted HPCD-treated protein extract (λ_max_ = 319 nm). However, fluorescence intensity decreased for HPCD and TS-treated extracts. Liu *et al*.^28^ also observed a decrease in intensity fluorescence in a protein purified solution from oriental sweet melon with PPO activity after ultrasonication process. These authors explained that the change in intensity could be due to complicated structural changes induced by ultrasound^28^. Regarding HPCD effect on fluorescence spectroscopy of HPCD-treated enzymes, Hu *et al*.^4^ reviewed different effects on fluorescence intensity reported in the literature related to structural change according to its origin and environment. For instance,an increase in the relative fluorescence intensity of horseradish POD was observed after HPCD treatment and the maximum wavelength red-shifted, indicating a change of Trp surroundings to a more polar environment^29^. Li *et al*.^30^ also observed a red-shifted in the maximum wavelength and a decrease of the fluorescence intensity after HPCD treatment in thaumatin like protein with high PPO activity. These authors attributed this behaviour to the development of a more polar environment and lower values of pH due to the formation of bicarbonate, carbonate and H^+^.

Although no POD activity was found in the protein extract before treatment, the interpretation of the data is very complicated since, although the obtained protein extract presented high PPO activity, it also contained a protein fraction co-extracted with the PPO.

**Figure 6:**
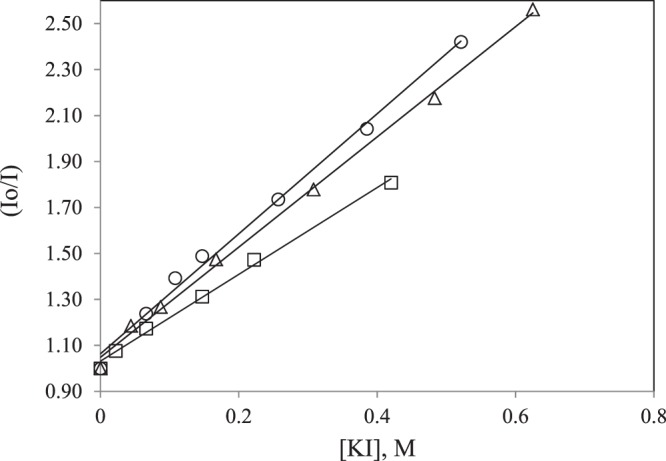
Stern-Volmer plot (see Eq. 3). Ratio of fluorescence intensities in the absence and presence of a given concentration of the iodide quencher (I_o_/I), for protein extracts with PPO activity (□) after HPCD treatment (○) and after TS treatment (Δ).

Quenching studies of the protein extract with PPO activity by KI were performed to examine the different location of tryptophan residues in untreated and HPCD and TS treated extracts. In this work a linear Stern-Volmer plot (Eq. 3) was obtained and, from the slopes, the Stern-Volmer quenching constants, K_SV_, for the untreated and HPCD and TS-treated PPO extracts were obtained (Table 2 and Fig. 6). Higher values of the slope, K_SV_, indicate a greater exposure of the fluorophore to the quencher^17^. An ANOVA was performed to test if there were any statistically significant differences among the slopes of Eq. 3, the Stern-Volmer quenching constants, for the non-treated and HPCD and TS treated extracts at the 95% confidence level. The lowest value for K_SV_ was obtained for the untreated protein extract, while the HPCD-treated extract presented the highest K_SV_ value. The different values obtained for the untreated and treated extract confirms conformational changes on proteins in the extract since fluorophore accessibility was affected.

**Table 2:** Terms of regression to the Stern-Volmer equation (Eq. 3).

protein extract	K_SV_, M^−1^	Intercept	R^2^
Non-treated	1.889 ± 0.061	1.031 ± 0.012	0.9980
HPCD-treated	2.609 ± 0.091	1.064 ± 0.025	0.9969
US-treated	2.395 ± 0.056	1.049 ± 0.018	0.9987

## Conclusions

In the present study, PPO from ‘*Golden Delicious*’ apple was concentrated by reversed micellar extraction. At the best extraction conditions PPO activity recovery was 99% and purification fold around 17 from a crude extract with high PPO activity. This extract was treated by HPCD and TS treatments achieving low residual activity of PPO. It has been observed that the protein fraction after treatment presented a greater exposure of the fluorophore to the quencher, although no convinced explanation of the PPO inactivation mechanism can be stablished since reversed micellar extraction is a concentration technique and PPO could not be isolated.

## References

[1] Briongos H., Illera A.E., Sanz M.T., Melgosa R., Beltrán S., Solaesa A.G. (2016). Effect of high pressure carbon dioxide processing on pectin methylesterase activity and other orange juice properties. LWT.

[2] Illera, A. E. *et al*. Evaluation of HPCD batch treatments on enzyme inactivation kinetics and selected quality characteristics of cloudy juice from Golden delicious apples. *Journal of Food Engineering* 258810 (2018).

[3] Illera A.E., Sanz M.T., Benito-Román O., Varona S., Beltrán S., Melgosa R., Solaesa A.G. (2018). Effect of thermosonication batch treatment on enzyme inactivation kinetics and other quality parameters of cloudy apple juice. Innovative Food Science & Emerging Technologies.

[4] Hu W, Zhou L, Xu Z, Zhang Y, Liao X (2013). Enzyme Inactivation in Food Processing using High Pressure Carbon Dioxide Technology. Critical Reviews in Food Science and Nutrition.

[5] Illera AE, Sanz MT, Beltrán S (2019). The Journal of Supercritical Fluids High pressure CO_2_ solubility in food model solutions and fruit juices. The Journal of Supercritical Fluids.

[6] Kadkhodaee R, Povey MJW (2008). Ultrasonic inactivation of Bacillusα-amylase. I. effect of gas content and emitting face of probe. Ultrasonics Sonochemistry.

[7] Imm JY, Kim SC (2009). Convenient partial purification of polyphenol oxidase from apple skin by cationic reversed micellar extraction. Food Chemistry.

[8] Mathew DS, Juang RS (2007). Role of alcohols in the formation of inverse microemulsions and back extraction of proteins/enzymes in a reverse micellar system. Separation and Purification Technology.

[9] Pires MJ, Cabral JMS (1996). Liquid - Liquid Extraction of Proteins with Reversed Micelles. Biotechnol. Prog..

[10] Zhou P, Smith NL, Lee CY (1993). Potential Purification and Some Properties of Monroe Apple Peel Polyphenol Oxidase. Journal of Agricultural and Food Chemistry.

[11] Rocha AMCN, Morais AMMB (2001). Characterization of polyphenoloxidase (PPO) extracted from ‘Jonagored’ apple. Food Control.

[12] Noh KH, Imm JY (2005). One-step separation of lysozyme by reverse micelles formed by the cationic surfactant, cetyldimethylammonium bromide. Food Chemistry.

[13] Soysal Ç, Söylemez Z, Bozoǧlu F (2004). Effect of high hydrostatic pressure and temperature on carrot peroxidase inactivation. European Food Research and Technology.

[14] Liu JG, Xing JM, Chang TS, Liu HZ (2006). Purification of nattokinase by reverse micelles extraction from fermentation broth: Effect of temperature and phase volume ratio. Bioprocess and Biosystems Engineering.

[15] Benito-Román, Sanz, M. T., Illera, A. E., Melgosa, R. & Beltrán, S. Polyphenol oxidase (PPO) and pectin methylesterase (PME) inactivation by high pressure carbon dioxide (HPCD) and its applicability to liquid and solid natural products. *Catalysis Today* article in press, 10.1016/j.cattod.2018.12.051.

[16] Benito-Román (2019). Pectin methylesterase inactivation by High Pressure Carbon Dioxide (HPCD). Journal of Supercritical Fluids.

[17] Möller M, Denicola A (2002). Protein tryptophan accessibility studied by fluorescence quenching. Biochemistry and Molecular Biology Education.

[18] Janovitz-Klapp A, Richard F, Nicolas J (1989). Polyphenoloxidase from apple, partial purification and some properties. Phytochemistry.

[19] Qiang L, Hongyan C, Kuanhong L, Yajun S (1998). Biochemical Engineering Journal. Biochemical Engineering Journal.

[20] Dekker, M. *et al*. Effect of temperature on the reversed micellar extraction of enzymes. *The Chemical Engineering Journal* **46** (1991).

[21] Chen YL, Su CK, Chiang BH (2006). Optimization of reversed micellar extraction of chitosanases produced by Bacillus cereus. Process Biochemistry.

[22] Soni K, Madamwar D (2000). Reversed micellar extraction of an extracellular acid phosphatase from fermentation broth. Process Biochemistry.

[23] Yu YC, Chu Y, Ji JY (2003). Study of the factors affecting the forward and back extraction of yeast-lipase and its activity by reverse micelles. Journal of Colloid and Interface Science.

[24] Marrufo-Hernández NA, Palma-Orozco G, Beltrán HI, Nájera H (2017). Purification, partial biochemical characterization and inactivation of polyphenol oxidase from Mexican Golden Delicious apple (Malus domestica). Journal of Food Biochemistry.

[25] Terefe NS (2009). The kinetics of inactivation of pectin methylesterase and polygalacturonase in tomato juice by thermosonication. Food Chemistry.

[26] O’Donnell CP, Tiwari BK, Bourke P, Cullen PJ (2010). Effect of ultrasonic processing on food enzymes of industrial importance. Trends in Food Science and Technology.

[27] Jiang L (2014). Effects of ultrasound on the structure and physical properties of black bean protein isolates. Food Research International.

[28] Liu S (2017). Effect of ultrasonic processing on the changes in activity, aggregation and the secondary and tertiary structure of polyphenol oxidase in oriental sweet melon (Cucumis melo var. makuwa Makino). Journal of the Science of Food and Agriculture.

[29] Gui F (2006). Inactivation and structural change of horseradish peroxidase treated with supercritical carbon dioxide. Food Chemistry.

[30] Li R, Wang Y, Hu W, Liao X (2014). Changes in the activity, dissociation, aggregation, and the secondary and tertiary structures of a thaumatin-like protein with a high polyphenol oxidase activity induced by high pressure CO2. Innovative Food Science and Emerging Technologies.

